# Complete Genome Sequence of *Staphylococcus aureus* FCFHV36, a Methicillin-Resistant Strain Heterogeneously Resistant to Vancomycin

**DOI:** 10.1128/genomeA.00893-15

**Published:** 2015-08-13

**Authors:** John Anthony McCulloch, Alessandro Conrado de Oliveira Silveira, Aline da Costa Lima Moraes, Paula Juliana Pérez-Chaparro, Manoella Ferreira Silva, Lara Mendes Almeida, Pedro Alves d’Azevedo, Elsa Masae Mamizuka

**Affiliations:** a Department of Clinical and Toxicological Analyses, Faculty of Pharmaceutical Sciences, University of São Paulo, São Paulo, Brazil; b Department of Pharmaceutical Sciences, Regional University of Blumenau, Blumenau, Santa Catarina, Brazil; c Department of Plant Biology, Biology Institute, State University of Campinas, Campinas, São Paulo, Brazil; d Department of Periodontology, Dental Research Division, University of Guarulhos, Guarulhos, São Paulo, Brazil; e Department of Microbiology and Parasitology, Universidade Federal de Ciências da Saúde de Porto Alegre, Porto Alegre, Rio Grande do Sul, Brazil

## Abstract

We report here the sequence of the entire chromosome of *Staphylococcus aureus* strain FCFHV36, a methicillin-resistant strain heterogeneously intermediate to vancomycin, bearing a type II staphylococcal chromosome cassette *mec* element (SCC*mec*), belonging to multilocus sequence type (MLST) 105, and isolated from a vertebra of a patient with osteomyelitis.

## GENOME ANNOUNCEMENT

We present here the sequence of the entire chromosome of *Staphylococcus aureus* strain FCFHV36, recovered from a vertebral biopsy sample from a patient diagnosed with community-acquired osteomyelitis and under medical care in a hospital in the state of Santa Catarina, Brazil. FCFHV36 presents an MIC of vancomycin of 2 µg/ml, which classifies it as being susceptible to this antibiotic. The patient, however, did not respond to therapy with vancomycin. A heterogeneous resistance (heterogeneous vancomycin-intermediate *S. aureus* [hVISA]) profile was detected by the population analysis profile-area under the curve (PAP-AUC) technique (1), meaning that cell subpopulations of this strain present higher MICs than those of the overall cell population. The PAP-AUC ratio of FCFHV36 to hVISA type strain Mu3 was 1.02.

The total genomic DNA of FCFHV36 was used to construct a paired-end (PE) library and a mate-paired (MP) library, which were separately sequenced using the MiSeq platform (Illumina, Inc.). A total of 4,981,028 75-bp-long reads were generated for the paired-end library, and a total of 1,181,570 250-bp-long reads were generated for the mate-paired library, which had a mean insert distance of 2,700 bp. Both libraries were simultaneously used as input for *de novo* assembly using the A5 pipeline (2), generating 46 contigs (sum, 2.84 Mbp; *N*_50_, 147.9 kbp; max length, 385.9 kbp). In order to build a scaffold, the contigs were ordered by synteny against a reference chromosome using Gepard (3). The reference genome chosen was the publicly available genome presenting the most similar *k*-mer spectrum to the contigs, as determined using KmerFinder (4), which was *S. aureus* JH1 (NCBI GenBank accession no. NC_009632). Contigs pertaining to plasmids were separated. The contig order was verified by aligning mate-paired reads against the scaffold and verifying the existence of mate pairs straddling the gap close to the mean insert distance using Geneious 7 (5). The correct position of contigs without synteny to the JH1 reference genome was also determined by mate-paired distance information. Gaps were then filled with GapFiller using reads from the paired-end library. After manual curation of gaps, the final circularized chromosome was annotated with Prokka (6), and features were manually curated by blasting against the GenBank nr database. Insertion sequences were found using the ISfinder database (7) and annotated manually using Artemis (8).

The chromosome of FCFHV36 carries 2,619 protein-coding sequences, 7 pseudogenes, 58 tRNA genes, and 16 rRNA genes. The *mecA* gene, which confers resistance to most β-lactams, is carried by a type II staphylococcal chromosome cassette *mec* element (SCC*mec*). *In silico* multilocus sequence typing (MLST) was able to attribute sequence type 105 (ST105) to the strain. Comparative genomics between this sequence and vancomycin-susceptible, VISA, and other hVISA stains will help determine the polymorphisms that correlate with decreased vancomycin susceptibility in *S. aureus*, which is hard to detect in the clinical laboratory.

### Nucleotide sequence accession number.

The complete genome sequence of *S. aureus* strain FCFHV36 has been deposited in DDBJ/EMBL/GenBank under the accession no. CP011147.
